# Diphtheria in Boston, Erie County, N. Y.

**Published:** 1862-11

**Authors:** 


### DIPHTHERIA IN BOSTON, ERIE COUNTY, N. Y.

We learn from Dr. S. S. Davis that diphtheria is now, and has been for some time, prevailing quite extensively, and in very severe and fatal form in Boston, Erie County, in the very district where typhoid fever was first recognized in western New York, by Prof. Austin Flint, formerly of this City. This was nearly twenty years ago, previous to which time continued fever was generally, perhaps universally regarded and treated as typhus; the antiphlogistic plan of treatment was adopted. Mercurials, emetics, cathartics and sometimes venæsection employed. Prof. Flint recommended and introduced the rational and universally adopted treatment of the present day, though not without considerable opposition. Dr. Davis regarded it as worthy of note that diphtheria should prevail so extensively, in low typhoid form, in the same locality, where a few years since, typhoid fever first made its appearance, or was first recognized in western New York.

